# Evaluation of Different Mode of Action Insecticides for the Control of *Bemisia tabaci*; Enhancement of Pesticide Efficacy

**DOI:** 10.3390/insects15110907

**Published:** 2024-11-20

**Authors:** Jackie Dunn, Debbie Ann Collins, Neil Audsley

**Affiliations:** Fera Science Ltd., York BioTech Campus, Sand Hutton, York YO41 1LZ, UK; jackie.dunn@fera.co.uk (J.D.); debbbie.collins@fera.co.uk (D.A.C.)

**Keywords:** whitefly, resistance, spinosad, PREV-AM, botanical, biopesticide

## Abstract

*Bemisia tabaci* (Gennadius) is a major pest worldwide, causing damage to a vast range of plants through its feeding on phloem sap and its vectoring of >100 plant viruses. Although not established in the UK, it is regularly introduced on planting material, which poses a significant plant health risk. Restrictions on pesticide use and increasing resistance to available active ingredients limit options for effective control of potential outbreaks. Alternative management options are required to mitigate this risk. There was high variability in the efficacy of the different modes of action products tested against two life stages (adults and larvae) as well as the Middle East–Asia Minor 1 (MEAM1) and Mediterranean (MED) cryptic species of *B. tabaci*. For both adults and larvae, MEAM1 were more susceptible than MED insects, possibly due to differences in resistance developed against some active ingredients. All products tested were effective to varying degrees against MEAM1 adults with Tracer (spinosad), PREV-AM (orange oil), Sequoia (sulfoxaflor), and FLiPPER (fatty acids) having similar efficacies (59–78% mortality). In contrast, PREV-AM and FLiPPER were most effective against MED adults (74% and 65% mortalities, respectively). Both MED and MEAM1 larvae were highly susceptible to FLiPPER and PREV-AM (>95% mortality), and the efficacy of Tracer and FLiPPER can be enhanced by using in combination with PREV-AM, and this can be achieved by using low doses of each product. Synergy was measured between PREV-AM and Tracer against MEAM1 larvae, which has the potential to provide effective control with a reduced pesticide application.

## Simple Summary

The sweet potato whitefly (*Bemisia tabaci*) is a major pest worldwide, primarily due to plant viruses (>100) it vectors. It is regularly introduced on plants imported into the UK and is, therefore, a plant health risk. Controlling this pest is challenging due to restrictions on use and resistance to available pesticides, so alternative management options are required to mitigate its risk. Available products with different modes of action were tested against adult and larval life stages of *B. tabaci* with high variability in their effectiveness. Two biopesticides, PREV-AM and FLiPPER, were highly effective against larval stages but less effective against adults. When PREV-AM was used in combination with the biopesticide Tracer against whitefly larvae, the efficacy of Tracer could be enhanced, and synergy between the two products, which were used at less than field-rate amounts, was measured. Hence, the combined application of Tracer and PREV-AM can potentially provide effective control of *B. tabaci* larvae and contribute towards reducing pesticide use.

## 1. Introduction

The sweet potato whitefly, *Bemisia tabaci* (Gennadius) (Hemiptera: Aleyrodidae), is a major pest of economically important crops worldwide. *Bemisia tabaci* causes damage by feeding on phloem sap and secretes honeydew, providing a substrate for the growth of sooty moulds. It is also a vector of >100 plant viruses, including the tomato yellow leaf curl virus, which is one of the most damaging viruses of tomato crops worldwide [1,2].

*Bemisia tabaci* is considered a complex of cryptic species [3]. The Middle East–Asia Minor 1 (MEAM1) species (formerly B-biotype) and the Mediterranean (MED) species (formerly Q biotype) are the most invasive and damaging species in the *B. tabaci* complex around the world. The MEAM1 species is an aggressive coloniser and an effective vector of viruses, whereas the MED species characteristically shows strong resistance to insecticides [2,4].

Although not strong flyers, adult *B. tabaci* can be dispersed long distances by the wind. All life stages are also carried on planting material and cut flowers of host species. The international trade in poinsettia plants for planting is considered to have been a major means of dissemination of MEAM1 species of *B. tabaci* within the EPPO region [5].

This insect has not established in the UK and is a notifiable pest subject to a policy of containment or eradication if found [6]. However, *B. tabaci* is regularly introduced to the UK on planting material, and there are outbreaks in ornamental crops, especially poinsettias, every year, which poses a significant threat as a vector of plant viruses in protected edible plants, including tomatoes, peppers and cucumbers. Infestations of *B. tabaci* are primarily controlled through the use of chemical pesticides, and although there are several active ingredients used in the UK for treating *B. tabaci* outbreaks [7], these outbreaks are still extremely difficult to control with the tools available. Furthermore, the increasing chemical resistance shown by *B. tabaci* to many pesticide groups has reduced pesticide effectiveness and is considered a major factor driving the establishment and spread of this invasive species [8,9,10]. Resistance to neonicotinoids and pyrethroids is particularly widespread but does vary from region to region, depending on use [2,11]. Neonicotinoids have been used extensively against *B. tabaci* due to their favourable properties against sap-feeding insects [12], leading to the first reported resistance in 1996 [13].

In Europe, the restriction on the use of many active ingredients, including neonicotinoids, has further reduced the options for control. This poses challenges for the effective eradication of incursions and outbreaks of *B. tabaci* should they occur in Great Britain. Hence, alternative control options (e.g., pesticides with different modes of action) or means to negate the resistance (e.g., inhibiting enzymes associated with metabolic resistance) developed by both MED and MEAM1 *B. tabaci* to some active ingredients are required so that incursions of *B. tabaci* can be adequately controlled.

The aims of this study were to identify alternative products for the control of *B. tabaci* and determine whether combinations of products could be used to enhance efficacy, reduce the amount of pesticide required and/or improve control. Products were chosen based on their efficacy against whiteflies or approval for use on protected crops in the UK.

## 2. Materials and Methods

### 2.1. Insects

Both MEAM1 and MED species of *B. tabaci* were from a laboratory culture maintained under quarantine conditions in Perspex^®^ cages (60 × 60 × 80 cm, Perspex Distribution Ltd., Blackburn, UK) on poinsettia (*Euphorbia pulcherrima* c.v. Lilo Pink) plants at 23 ± 1 °C; 16:8-h Light:Dark, following the method of Cuthbertson et al. [14]. Laboratory cultures of both MED and MEAM1 species were established >10 years from intercepted adult/pupal *B. tabaci* specimens received from the UK Plant Health and Seeds Inspectorate, and the two species were discriminated using established molecular techniques [15].

### 2.2. Pesticides (Formulated Products)

Formulated products tested against adult *B. tabaci* are listed in Table 1. All pesticides were purchased from East Riding Horticulture Ltd., York, UK, except PREV-AM, which was purchased from Oro Agri International Palmela, Portugal. Serial dilutions (×1, ×0.5, ×0.25, ×0.125, ×0.0625 of the field rate) of each pesticide were first prepared using distilled water as a diluent and dose-response data generated to determine ED_10_ (effective dose that corresponds to a 10% mortality relative to control mortality) concentrations. The ED_10_ concentrations were then used to test whether PREV-AM could enhance and potentially synergise the efficacy of other products (Tracer and FLiPPER).

### 2.3. Efficacy Testing

Efficacy testing on both larvae and adults was carried out following previously described methods [14]. Each replicate contained 10 insects, and all tests were repeated.

#### 2.3.1. Adults

The leaves on poinsettia plants were trimmed so that five leaves of good size and condition remained on each plant. Two plants were treated with each pesticide solution or water (control). Each leaf was dipped in its respective test (pesticide) solution or water (control) for 20 s and left to air dry.

Dose-response data were generated for each pesticide against the MEAM1 and MED species and used to generate ED_10_ values using Probit analysis [16].

Unsexed mixed-age adults of *B. tabaci* were added to clip cages, which were then attached to the treated leaves (the insects were on the underside of the leaf), with one clip cage on each leaf. The plants were covered with a ventilated bag and kept in a plant growth chamber (Fitotron, Sanyo Gallenkamp) at 25 °C, 65% RH and 16:8-h L:D. Insect mortality was assessed after 96 h, as previously described [14].

To assess potential synergy between products, methods were modified from Young et al. [17]. Whiteflies were first exposed to leaves treated with an ED_10_ of PREV-AM for 2 h before being transferred in clip cages to leaves treated with ED_10_ amounts of Tracer (PREV-AM/Tracer). PREV-AM and Tracer were also mixed (PREV-AM/Tracer mix) and applied together to test for potential synergy. An additional control was included to account for any handling mortality caused by transferring whiteflies to different plants. Insect mortality was assessed after 96 h.

#### 2.3.2. Larvae

Poinsettia plants were infested with *B. tabaci* by adding 10 adults within clip cages on the underside of the leaves for 48 h to lay eggs. The plants were kept in a plant growth chamber (Fitotron, Sanyo Gallenkamp, Leicestershire, UK) at 25 °C, 65% RH, and 16:8-h L:D throughout the experiments. After 48 h, the adults were removed, and the eggs were left to develop on the plants. After 12 days, the eggs had developed into second-instar larvae, the chosen development stage to test.

The leaves were then dipped in their respective test (pesticide) solution or water (control) for 10 s and left to air dry, as outlined by Cuthbertson et al. [14]. To assess potential synergism between products, leaves were either treated with an ED_10_ of PREV-AM, allowed to dry for 2 h, then treated with either an ED_10_ of Tracer or FLiPPER or the pesticide solutions were mixed together, leaves dipped for 10 s and left to air dry. Mortality was examined after seven days by monitoring larvae that had moulted into adults.

### 2.4. Statistical Analyses

Statistical analyses were performed using GraphPad Prism 9 for Windows. Percentage mortalities were compared between treatments using analysis of variance (ANOVA) followed by a post-hoc test (Tukey honestly significant difference test) to determine significant differences between treatments at the 5% level. Adjustments for control mortality, where necessary, were made using Abbott’s formula [18].

Synergistic interactions between products used in combination treatments were determined using Chi-square tests, as described by Ansari et al. [19].

## 3. Results

### 3.1. Bioassay Comparison

Preliminary tests assessed the comparative efficacy of leaf disc and intact leaf bioassays using the field rate amounts of Tracer against MEAM1 adults (Table 2). Results were comparable between bioassays; there was no significant difference between the two assay methods tested for both the control treatments and the efficacy of Tracer (*p* > 0.05).

### 3.2. Adult Stage

#### 3.2.1. Field Rate Pesticide Application

**Figure 1:**
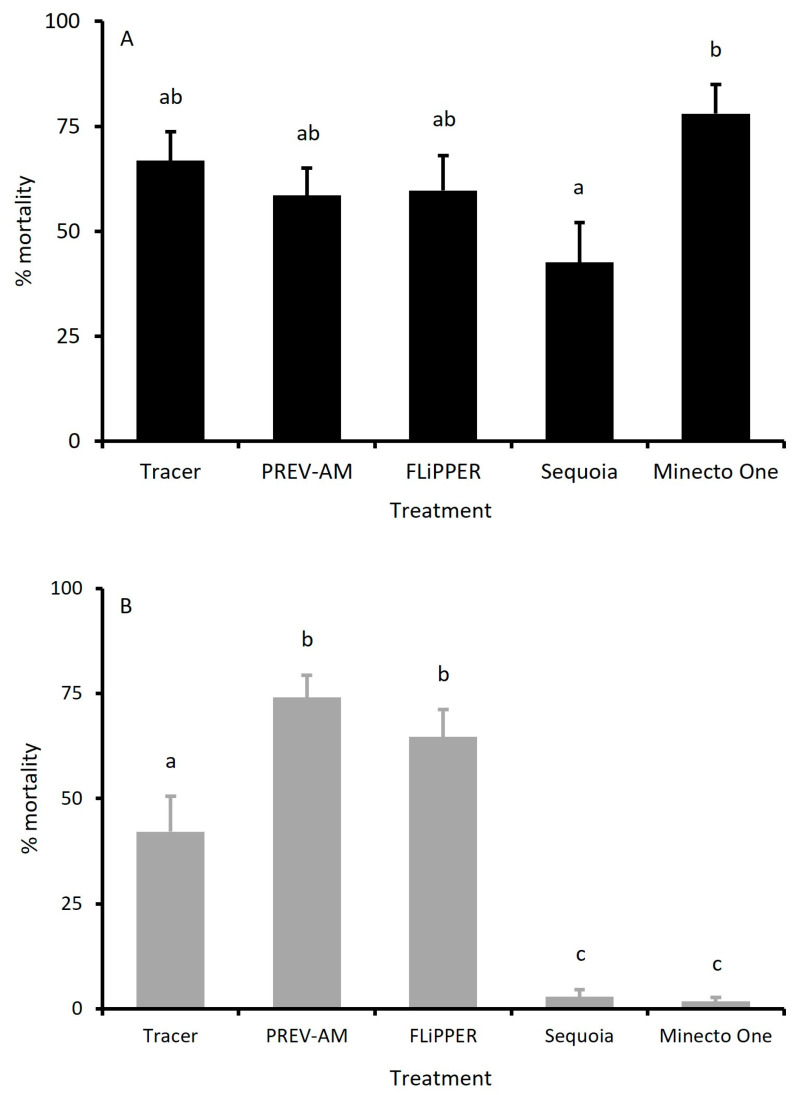
A comparison between field rate doses of pesticides on the apparent mortality rates of adult MEAM1 (**A**) and MED (**B**) *Bemisia tabaci* 96 h post-treatment. Mean ± S.E., n = 9–10. Different letters indicate significant differences between treatments (*p* < 0.05, Tukey’s test).

When tested against adults of the MEAM1 species, predicted % mortalities ranged from *c*. 53 to 83% 96 h post-treatment, compared to control mortalities of *c*. 20%. All products tested were significantly different from controls (*p* < 0.05) but not from each other, except for Sequoia and Minecto One (Figure 1A).

Figure 1B compares treatments against the adults of the MED species 96 h post-treatment. Sequoia and Minecto One were ineffective, whereas PREV-AM, FLiPPER, and Tracer-induced mortality (*c*. 50–79%) was significantly above the untreated 19.5% control mortality (*p* < 0.05). In terms of pairwise comparisons, Tracer was found to cause mortality significantly below (*p* < 0.05) that was observed with PREV-AM and FLiPPER; the latter two were identified as statistically equivalent (*p* > 0.05).

#### 3.2.2. Dose-Response Evaluation of Tracer and PREV-AM Against Adult *Bemisia tabaci* and Combined Effects

**Figure 2:**
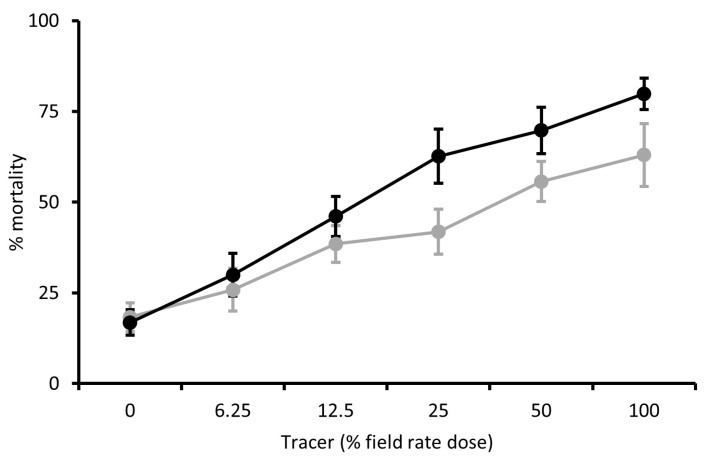
Mortality of adult MEAM1 (black line) and MED (grey line) *Bemisia tabaci* 96 h post-treatment with different doses of Tracer (% field rate dose). Mean ± S.E., n = 10–15.

The efficacy of different doses of Tracer against MED and MEAM1 adults of *B. tabaci* was dose-dependent (Figure 2). Using Probit analysis, the ED_10_ values calculated were 0.0058 g a.i./L (0.08 × field rate) for MED adults and 0.0018 g a.i./L (0.025 × field rate) for MEAM1 adults.

**Figure 3:**
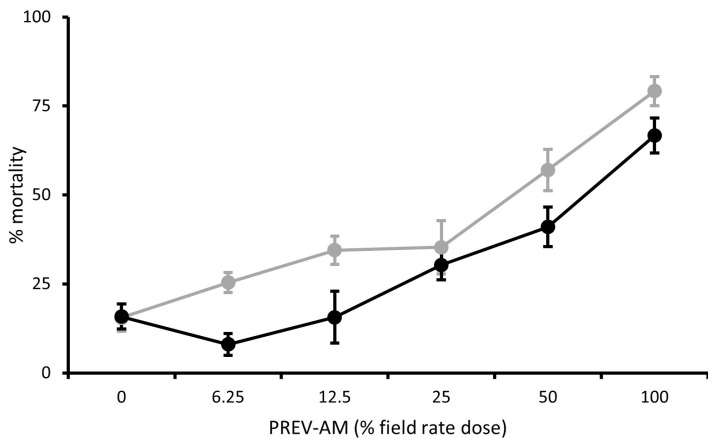
Mortality of adult MEAM1 (black line) and MED (grey line) *Bemisia tabaci* 96 h post-treatment with different doses of PREV-AM (% field rate dose). Mean ± S.E., n = 10–15.

Similarly, the efficacy of PREV-AM tested against adult *B tabaci* MED and MEAM1 species was also dose-dependent, as shown in Figure 3. The ED_10_ values calculated by Probit analysis were 0.0032 g a.i./L (0.08 × field rate) and 0.0092 g a.i./L (0.23 × field rate) for MED and MEAM1 species, respectively.

When the respective ED_10_ concentrations of Tracer and PREV-AM were tested in combination against MED and MEAM1 adults of *B. tabaci*, the apparent mortality was not significantly different to the additive effects of the individual treatments, demonstrating no enhancement of the efficacy of products.

### 3.3. Immature Stages

#### 3.3.1. Field Rate Application

##### MEAM1 Larvae

When tested against second-stage *B. tabaci* MEAM1 larvae, the field rate doses of FLiPPER and PREV-AM both induced >95% mortality and were not significantly different from each other (*p* < 0.05).

**Figure 4:**
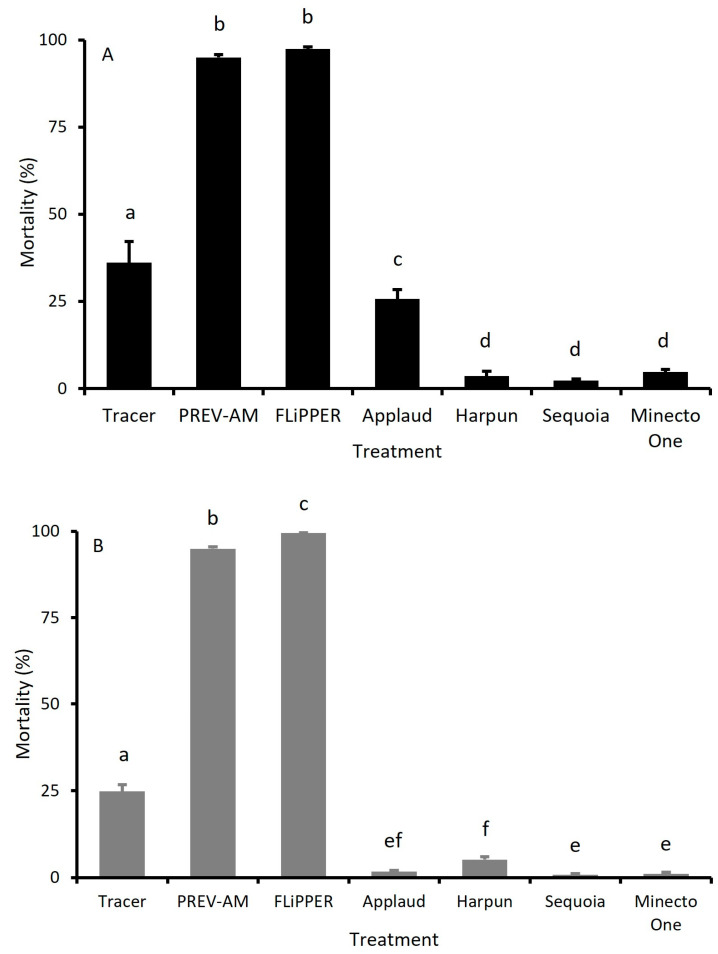
A comparison between field rate doses of pesticides on the survival (% mortality) *Bemisia tabaci* MEAM1 (**A**) and MED (**B**) second-stage larvae, means ± S.E., n = 10. Letters indicate significant differences between treatments (*p* < 0.05, Tukey’s test).

Tracer and Applaud resulted in mortalities of *c*. 36% and 26% mortality, respectively, which were significantly greater than controls (no mortality). In contrast, Harpun, Minecto-One and Sequoia were much less effective (<10% mortality) and not significantly different from each other or controls (Figure 4A).

##### MED Larvae

Mortalities of second-stage *B. tabaci* MED larvae treated with field rate doses of FLiPPER and PREV-AM were *c*. 95% and 99.5%, respectively, whereas Tracer only caused 25% mortality, and each of the three treatments was significantly different from each other (Figure 4B). Harpun had a minor effect (5% mortality), which was significantly different to controls (no mortality). Applaud, Sequoia, and Minecto One were largely ineffective and not significantly different from each other or the controls (Figure 4B).

#### 3.3.2. Dose-Response Evaluation of Tracer, PREV-AM and FLiPPER Against *Bemisia tabaci* Larvae

**Figure 5:**
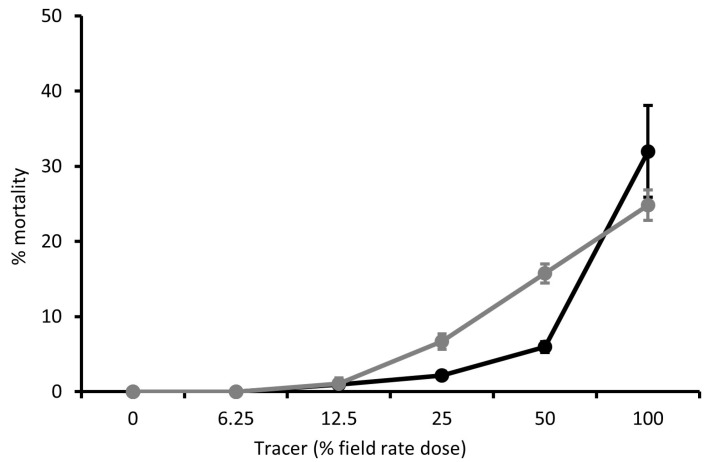
The dose-response effect of Tracer on mortality (percentage means) of *Bemisia tabaci* MEAM1 (black line) and MED (grey line) second-stage larvae, means ± S.E., n = 10.

**Figure 6:**
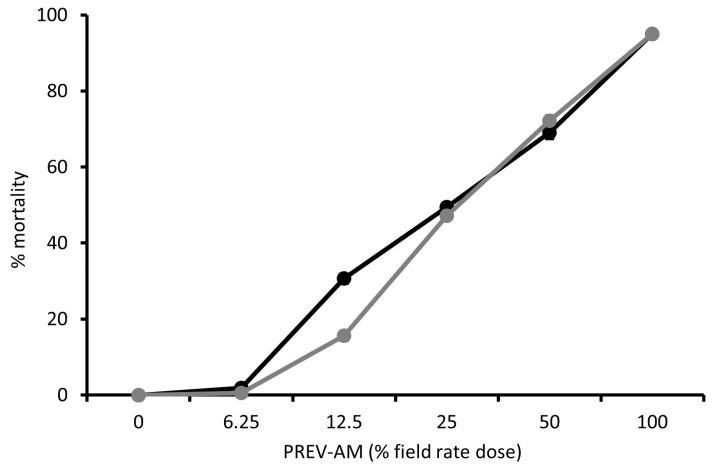
The dose-response effect of PREV-AM on mortality (percentage means) of *Bemisia tabaci* MEAM1 (black line) and MED (grey line) second-stage larvae, means ± S.E., n = 10.

**Figure 7:**
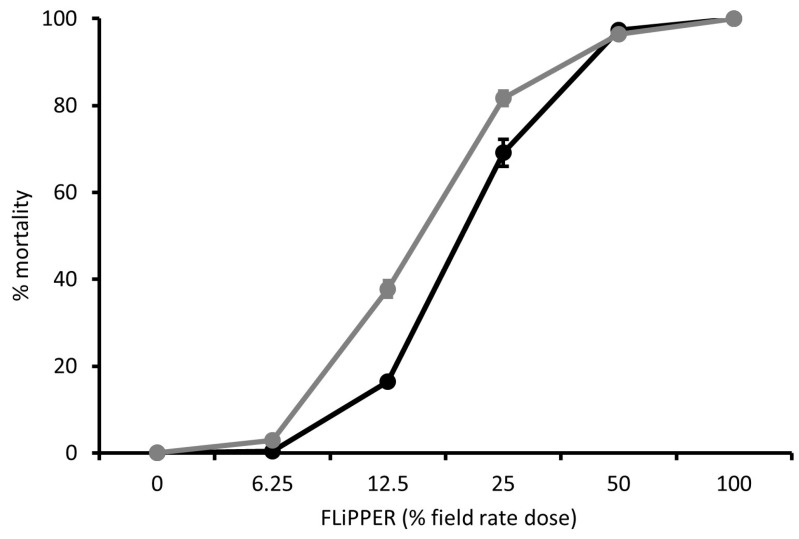
The dose-response effect of FLiPPER on mortality (percentage means) of *Bemisia tabaci* MEAM1 (black line) and MED (grey line) second-stage larvae; means ± S.E.; n = 10.

Dose-response curves were produced for Tracer (Figure 5), PREV-AM (Figure 6), and FLiPPER (Figure 7) against MEAM1 and MED *B. tabaci* larvae so that ED_10_ values could be established for use in tests for potential synergy between products.

The ED_10_ values generated by Probit analysis of dose-response data are shown in Table 3.

The field rate dose of FLiPPER was slightly phytotoxic to poinsettia; however, this biopesticide was equally as effective at a 50% field rate dose, where no phytotoxicity was observed.

#### 3.3.3. Combined Effects of Tracer and PREV-AM

##### MEAM1 Larvae

**Figure 8:**
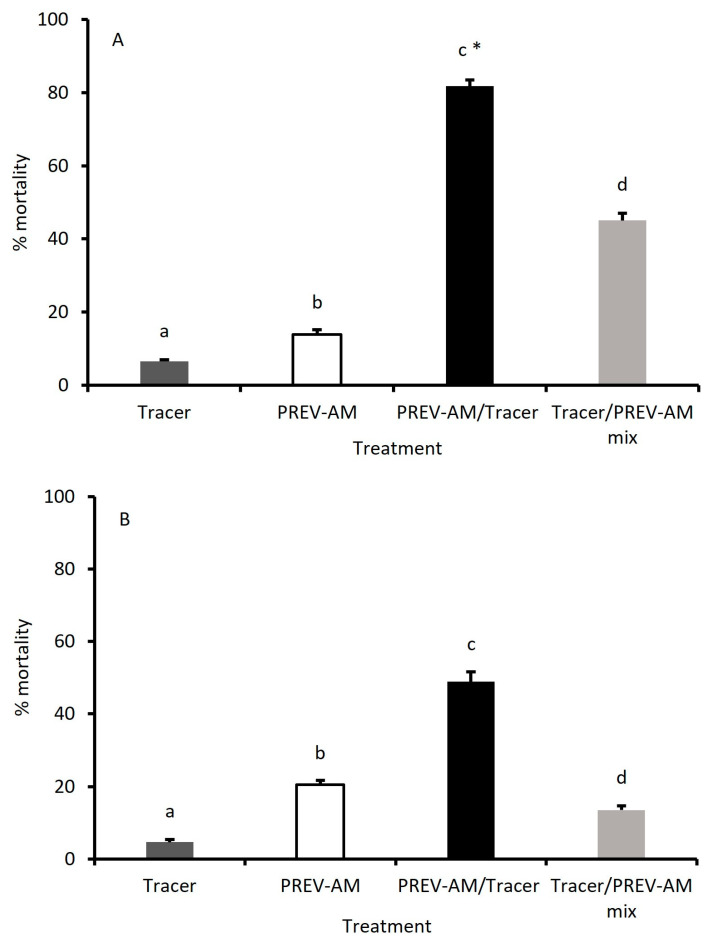
The mortality of *Bemisia tabaci* MEAM1 (**A**) and MED (**B**) second-stage larvae treated with ED_10_ doses of Tracer and/or PREV-AM, applied either separately (dark-grey and open bars), combined as two separate treatments (PREV-AM/Tracer; black bar) or as a mixture (Tracer/PREV-AM mix; light-grey bar). Means ± S.E., n = 10. All treatments are significantly different from each other, *p* < 0.05, as indicated by letters). The * indicates synergistic interaction between products in combination treatments determined using X^2^ tests (*p* < 0.05).

Figure 8A shows the mortality of MEAM1 larvae treated with ED_10_ doses of Tracer and PREV-AM either alone or in combination. All treatments are significantly different from each other (*p* < 0.05). Control survival was 100%. When used in combination, the ED_10_ doses of PREV-AM/Tracer (PREV-AM treatment followed by Tracer treatment) caused a four-fold increase in mortality to 81.2% compared to the additive mortalities of single treatments of Tracer and PREV-AM (20.3%), resulting in a significant synergistic increase in mortality (*p* < 0.05). Although treatment with a mixture of Tracer and PREV-AM resulted in a 2.22-fold increase in mortality to 45.1% and exceeded the additive effects of Tracer and PREV-AM, this increase was not synergistic (*p* > 0.05).

##### MED Larvae

The mortality of MED larvae treated with ED_10_ doses of Tracer and PREV-AM used alone and in combination is shown in Figure 8B. All treatments are significantly different from each other (*p* < 0.05). Control mortality was zero.

The additive mortality of ED_10_ doses of Tracer and PREV-AM is 25.2%, which increases 1.9 times to 48.9% when PREV-AM treatment is followed by Tracer, but this was not significantly greater than the additive treatment. The mortality caused by the mixture of Tracer/PREV-AM was only 13.5%.

#### 3.3.4. Combined Effects of PREV-AM and FLiPPER

##### MEAM1 Larvae

**Figure 9:**
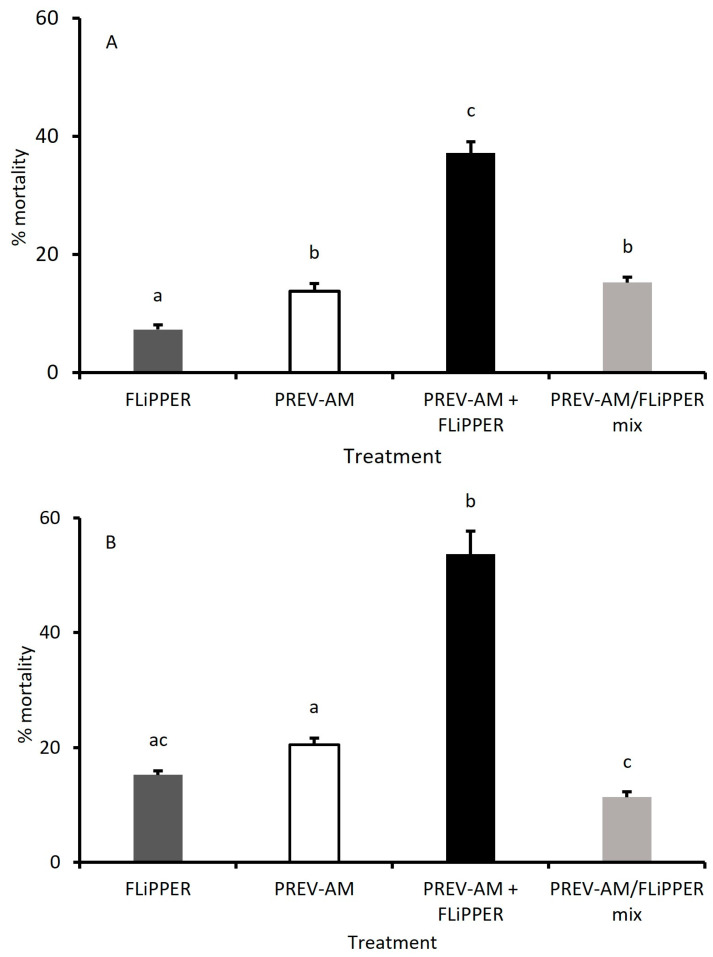
The mortality of *Bemisia tabaci* MEAM1 (**A**) and MED (**B**) second-stage larvae treated with ED_10_ doses of FLiPPER and/or PREV-AM, applied either separately (dark-grey and open bars), combined as two separate treatments (PREV-AM/FLiPPER; black bar) or as a mixture (PREV-AM/FLiPPER mix; light-grey bar). Means ± S.E., n = 10. Means with same letters are not significantly different (*p* < 0.05, Tukey’s test).

Figure 9A shows the mortality of MEAM1 larvae treated with ED_10_ doses of FLiPPER and PREV-AM either alone or in combination.

When used in combination, the 37.2% mortality caused by ED_10_ doses of PREV-AM/FLiPPER (PREV-AM treatment followed by FLiPPER treatment) was 1.76 times >, but not significantly different to the additive 21.2% mortality of FLiPPER plus PREV-AM treatments alone (*p* > 0.05). The FLiPPER/PREV-AM mix resulted in only 15.2% mortality, which is <the additive effects and not significantly greater than the mortality of PREV-AM alone. Control survival was 100%.

##### MED Larvae

The mortalities of MED larvae treated with ED_10_ doses of PREV-AM and FLiPPER alone and in combination are shown in Figure 9B. When used in combination, PREV-AM/FLiPPER (PREV-AM treatment followed by FLiPPER treatment) caused a significant (but not synergistic) increase in mortality to 53.7%, exceeding the additive effects (35.7% mortality) of PREV-AM + FLiPPER alone. Applying a mixture of FLiPPER/PREV-AM did not exceed the additive effects, nor the individual mortalities caused by PREV-AM + FLiPPER alone (Figure 9B). Control survival was 100%.

## 4. Discussion

The results of this study show that there is high variability in the efficacy of products against the two life stages tested (adults and larvae) as well as the different (MEAM1 and MED) cryptic species of *B. tabaci*. Both adults and larval MEAM1 were more susceptible than MED, which could be related to differences in resistance developed against some active ingredients (reviewed by Horowitz et al. [2]).

Spinosad, a contact or orally acting biopesticide derived from the soil bacterium *Saccharopolyspora spinosa* [20] tested using the formulated product Tracer, was most effective against MEAM1 adults, with lower efficacy against MED adults and both larval species when applied at field rate doses. However, mortalities were all lower than those reported by Kumar and Poehling [21], who tested spinosad in different life stages of *B. tabaci*. Foliar sprays of field-rate amounts of spinosad inhibited egg hatch by up to 80% and caused 100% mortality of pupae (fourth instar larvae). Efficacy against larvae was age- and dose-dependent, with 100% mortality achieved between 3 and 9 days post-treatment. They also reported that spinosad was highly persistent, with residual effects lasting at least 15 days in both the laboratory and greenhouse trials. Resistance monitoring in field strains of MEAM1 and MED *B. tabaci* from different regions in China determined low or no resistance to spinosad in the different populations tested [22,23]. The differences in the reported mortality rates may be due to the different experimental procedures used and/or levels of resistance in the different cryptic species and populations. Although neonicotinoids were not tested, any resistance to this group of pesticides may exhibit cross-resistance to spinosad, as demonstrated with neonicotinoid-resistant strains of *Leptinotarsa decemlineata* [24].

FLiPPER is a contact biopesticide comprising unsaturated fatty acids (C14–C20), which has a physical mode of action but can also penetrate cells to disrupt their functions. It is recommended for use against whiteflies, including *B. tabaci* [25]. *Bemisia tabaci* eggs and larvae have been used in laboratory and field trials to evaluate product efficacy under both protected and open field conditions. High efficacy was reported under all conditions tested [26], and Suma et al. [27] reported high efficacy of FLiPPER against *B. tabaci* eggs and larvae under laboratory conditions. Similar efficacies of field-rate doses using leaf-dip assays against both MEAM1 and MED larvae are reported in this study. Furthermore, dose-response data show that similar efficacy can be achieved with 50% of the field-rate dose, thereby reducing the amount of product required. However, FLiPPER was not as effective against either MED or MEAM1 adults. This may be a result of the product being applied directly onto larvae through dipping infested leaves, whereas pesticide application for adults was through contact with treated leaves.

PREV-AM (a.i., orange oil) is a biopesticide with a physical mode of action. It has previously been tested for efficacy against *B. tabaci* in Egypt under field conditions with efficacies of *c*. 55% in adults on cucumber [28], 46–89% in general populations (life stage not stated) on kidney bean [29], and 50–80% in adults [30,31] and 57–71% in larvae [30] on tomato. When MED *B. tabaci* adults were exposed to PREV-AM treated tomato plants under laboratory conditions, field rate and 50% field rate doses caused a significant (32% and 64%, respectively) reduction in the subsequent numbers of eggs laid and progeny reaching first, second, and third instars (up to 75% mortality compared to controls). A 10% field rate dose only had a significant effect on the number of larvae reaching the third instar [9]. This study shows that MEAM1 and MED larvae were equally susceptible to PREV-AM. Over a 3-day period, mortality was as high as 95%, which was greater than the reports described above, but this may be due to the different types of trials (laboratory versus field) carried out. In contrast, PREV-AM was not as effective in adults, causing an apparent 58% and 74% reduction in survival of MED and MEAM1 adults, respectively.

Sulfoxaflor (Sequoia) is a sulfoxamine compound and a nicotinic acetylcholine receptor competitive modulator and is approved for use in the UK on protected crops. It was largely ineffective against *B. tabaci,* only showing any efficacy against MEAM1 adults (*c*. 43% mortality). Similarly, when tested in the field on cantaloupe melons, Sequoia was reported to have inconsistent residual activity against adult whiteflies [32]. When tested against eggs and early-stage larvae of insecticide-susceptible whiteflies and compared to commercial products, sulfoxaflor was as potent as spirotetramat and imidacloprid but less potent than acetamiprid, thiamethoxam, and dinotefuran in laboratory studies targeting eggs and early-stage larvae. In field trials on multiple crops, the efficacy of sulfoxaflor (up to 87% mortality) against whitefly was similar to the neonicotinoids imidacloprid and thiamethoxam but less than acetamiprid, 12–16 days post-second treatment [33]. Low levels of resistance to sulfoxaflor have been reported in China [34].

The ryanodine receptor modulator, cyantraniliprole, is approved for use in the UK on outdoor and protected horticultural crops, including tomatoes and ornamentals. Only MEAM1 adults were susceptible to the formulated product Minecto One (>85% mortality), whereas mortality of MED adults and larvae, as well as MEAM1 larvae, was very low (≤5%). Variable levels of susceptibility and resistance to cyantraniliprole have been reported for populations of both MED and MEAM1 species (reviewed by Horowitz et al. [2]). In field experiments in Egypt, *B. tabaci* were highly susceptible to cyantraniliprole [35], and similar susceptibility was reported by Gouvêa et al. [36] for MEAM1 adults on tomato plants. However, medium to high resistance to cyantraniliprole has been reported in China, and in MED species, overexpression of cytochrome P450 genes was shown to be involved [37,38].

Two insect growth regulators, buprofezin (Applaud 25 SC) and pyriproxyfen (Harpun), were tested against both MED and MEAM1 larvae. Buprofezin is an inhibitor of chitin synthesis and is approved for use in the UK against *B. tabaci* on ornamental protected plants. When tested under laboratory conditions, it was ineffective against MED larvae and only caused 26% mortality in MEAM1 larvae. Pyriproxyfen is a juvenile hormone analogue which disrupts moulting and metamorphosis. It is approved for use against whiteflies on protected tomatoes in the UK. (Liaison), however, it had very limited activity (<6% mortality) on both MED and MEAM1 larvae under laboratory conditions. These data suggest that both buprofezin and pyriproxyfen would not be effective enough to use against outbreaks of *B. tabaci* in the UK.

Although IGRs were recommended for use in integrated management programmes for *B. tabaci*, their widespread use has led to resistance against both buprofezin and pyriproxyfen in many countries. Resistance was reported to be stronger in MED compared to MEAM1 biotypes (reviewed by Horowitz et al. [2]). This is reflected in the efficacy data for Applaud but not for Harpun, which was equally ineffective against both biotypes.

Both target-site and metabolic resistance to insecticides have been identified in *B. tabaci*. Resistance is widespread and developed against most insecticides used for *B. tabaci* control in both MED and MEAM1 species (reviewed by Horowitz et al. [2]). It is possible that the low efficacy of some of the products tested is in part due to levels of resistance in the populations of MED and MEAM1 species used in this study.

The enzymes responsible for metabolic resistance can be inhibited to enhance or recover the efficacy of insecticides (reviewed by Metcalf [39]). Piperonyl butoxide is a recognised inhibitor of mixed-function oxidases, as well as esterases associated with insecticide resistance. Piperonyl butoxide was shown to inhibit esterases in adult MEAM1 *B. tabaci* and, in field trials, enhance the efficacy of pyrethroids against this insect [40]. Botanicals have also been shown to increase the efficacy of pesticides [41]. When tested on *B. tabaci* larvae, PREV-AM was shown to have greater than additive effects when used in combination with spinosad and significant synergy was measured for MEAM1 larvae. It is possible that orange oil, the active ingredient of PREV-AM, is inhibiting enzymes that detoxify spinosad to enhance efficacy, although the direct effect on these enzymes was not measured. Pre-treatment with PREV-AM prior to spinosad is clearly important for inhibition because applying PREV-AM and spinosad (Tracer) together was not as effective. Pre-treatment most likely allowing time for optimal inhibition of enzyme activity, assuming this is PREV-AM’s mode of action. In comparison, Young et al. [40] demonstrated that pre-treatment with PBO restored effective control by pyrethroids of highly resistant adult MEAM1 *B. tabaci*.

Both PREV-AM and FLiPPER provide high levels of control of both MED and MEAM1 larvae, whereas other products would not achieve sufficient control when used alone. The efficacy of Tracer and FLiPPER can be enhanced by using them in combination with PREV-AM, and this can be achieved by using lower doses of each product. Synergy was measured between PREV-AM and Tracer against MEAM1 larvae, demonstrating the potential of providing effective control with reduced pesticide application. However, further work is required to optimise the timings of applications and doses required to significantly synergise efficacies for all combinations, as well as for other products.

## Figures and Tables

**Table 1:** List of pesticides, their active ingredients, and the field rate doses used to treat *Bemisia tabaci* adults and/or larvae.

Pesticide	Active Ingredient (a.i.)	Field Application Rate (g a.i./L)
Tracer	Spinosad	0.072
FLiPPER	Fatty Acids	7.68
PREV-AM	Orange oil	0.04
Harpun	Pyriproxyfen	0.075
Applaud	Buprofezin	0.132
Sequoia	Sulfoxaflor	0.048
Minecto One	Cyantraniliprole	0.074

**Table 2:** Comparison between controls and efficacy of Tracer against *Bemisia tabaci* MEAM1 adults using leaf disc and intact leaf assays 96-h post-treatment; mean mortality ± S.E., n = 10.

Treatment	Mortality (%)	
	Leaf Disc	Intact Leaves
Control Tracer	18.95 ± 4.98 85.90 ± 2.69	14.75 ± 4.60 80.59 ± 4.77

**Table 3:** The ED_10_ values for Tracer, PREV-AM, and FLiPPER on *Bemisia tabaci* MEAM1 and MED larvae generated by Probit analysis of dose-response data.

Pesticide	*Bemisia tabaci* Species	ED_10_ Value (g a.i./L)
Tracer	MEAM1	0.037
	MED	0.029
PREV-AM	MEAM1	0.0033
	MED	0.0044
FLiPPER	MEAM1	0.837
	MED	0.568

## Data Availability

The data that support this study are available upon request from the authors.
